# How to... train your skills goes digital! A project report on the development and implementation of practice-oriented digital student tutorials

**DOI:** 10.3205/zma001526

**Published:** 2022-02-15

**Authors:** Maria Heimbach, Katharina Holzmann, Philipp Stein, Lea Stief, Pascal O. Berberat, Meike Dirmeier

**Affiliations:** 1 Technische Universität München, Fakultät für Medizin – Klinikum rechts der Isar, TUM Medical Education Center (TUM MEC), Lehrstuhl für Medizindidaktik, medizinische Lehrentwicklung und Bildungsforschung, München, Germany

**Keywords:** medical education, digitalisation, medical student, peer-assisted learning, model-based learning, practical skills, inverted classroom model, COVID-19, peer-teaching

## Abstract

**Objective: **This project report outlines the development and implementation of digital tutorials (“How to... train your skills goes digital!”) by peer tutors (TUTs) based on previously established in-person tutorials, as well as the subsequent combination of both approaches. The TUTs' objective, in spite of corona-related restrictions and strict hygiene requirements during the pandemic, was to provide fellow students with the opportunity to learn, practice and reflect on clinical-practical skills.

**Methodology: **In a collaborative undertaking, the TUTs first analyzed the learning objectives of the in-person tutorials in order to be able to design content-matched digital tutorials without entirely abandoning the practical aspect. The Moodle learning management platform was selected as the appropriate tool for delivery of the relevant theoretical knowledge. Practical exercises are embedded in the digital tutorials during online meetings. The participants (PTs) create their own models in the sense of a home skills station based on instructions provided via Moodle. Acceptance was systematically documented via questionnaires.

**Results: **The digital tutorials were well accepted by the PTs (n=64). Evaluation (response rate: 37.5%) outcomes were consistently positive. Both course implementation and the PTs' own progress were rated “good” to “very good”. Nevertheless, the PTs do not yet feel well-prepared to carry out the various activities practiced independently.

In the winter semester of 2020/21, the in-person tutorials were also reintroduced in a combined format. The marked demand for the tutorials may indicate the PT preference for practice on the simulation center models.

**Conclusion: **The systematic combination of digital and in-person tutorials using the flipped classroom approach would appear to make sense in the long run. The effectiveness and sustainability of this approach in comparison with in-person tutorials only should be further investigated.

## Zusammenfassung

**Zielsetzung: **Der vorliegende Projektbericht beschreibt die Entwicklung und Umsetzung digitaler Tutorien („How to... train your skills goes digital!“) durch studentische Tutor*innen (TUT) auf Grundlage bereits etablierter Präsenztutorien, sowie die anschließende Verschränkung beider Konzepte. Das Ziel der TUT war es hier, den Mitstudierenden in einer Phase der coronabedingten Ausgangsbeschränkungen und der intensivierten Hygieneauflagen dennoch die Möglichkeit zu geben, klinisch-praktische Fertigkeiten zu trainieren und zu reflektieren.

**Methodik:** In einem gemeinsamen Prozess wurden zunächst die Lerngegenstände der Präsenztutorien von den TUT analysiert, um inhaltlich kongruente digitale Tutorien konzipieren zu können, ohne dabei völlig auf den praktischen Aspekt verzichten zu müssen. Zur Vermittlung der theoretischen Kenntnisse wurde das Learning Management System Moodle als geeignetes Instrument identifiziert. Die praktischen Übungen erfolgen innerhalb der Umsetzung der digitalen Tutorien während virtueller Meetings. Hierfür erstellen die Teilnehmenden (TN) nach einer über Moodle bereitgestellten Anleitung selbst Modelle im Sinne einer Home Skills Station. Die Akzeptanz wurde mittels Fragebogen systematisch erfasst.

**Ergebnisse: **Die digitalen Tutorien werden bisher von den TN (n=64) gut angenommen. So zeigt ihre Evaluation (Rücklauf: 37,5%) durchweg positive Ergebnisse. Es werden sowohl die Kursdurchführung als auch der eigene Lernfortschritt durch die TN als gut bis sehr gut bewertet. Nichtsdestotrotz fühlen sich die TN auf die selbstständige Durchführung der jeweiligen Tätigkeiten noch nicht optimal vorbereitet.

Im Wintersemester 2020/21 wurden auch die Präsenztutorien unter einer Verschränkung wieder eingeführt. Die starke Nachfrage nach diesen lässt möglicherweise auf die Präferenz der TN schließen, an den Modellen des Simulationszentrums zu üben.

**Schlussfolgerung: **Die systematische Verschränkung von Digital und Präsenz im Sinne des Flipped Classroom Konzeptes erscheint langfristig sinnvoll. Die Wirksamkeit und Nachhaltigkeit im Vergleich mit der reinen Präsenz müsste weiter untersucht werden.

## Introduction

“How to... train your skills!” is a Medical Education Center (MEC) program at the Technical University of Munich (TUM) for training of clinical-practical skills by and with peer tutors (TUTs) [1], [2], [3]. It was established in the Medical Training Center (MTC) in the winter semester of 2019/20. Since curricular courses with medical lecturers for team and skills training have been predominantly held at the MTC till now, this optional program constitutes a unique offer. It was developed and implemented by the TUTs themselves at the request of the students. 

Given the provisional restrictions [4] during the Corona pandemic [5], “How to... train your skills” could not be held in the summer semester of 2020. The TUTs, who are also authors of this project report, developed “How to... train your skills goes digital!” to address this short-fall and offer their fellow students functional training in clinical-practical skills in this period of involuntary passivity and social distancing, while at the same time promoting a lively and motivating opportunity for exchange [6], [7]. To what extent this objective was achieved is critically assessed in the following.

## Concept and development

One particular challenge lay in designing a method for teaching practical skills without being able to rely on the infrastructure of the MTC with its simulators and models as well as the physical presence of instructors and learners [8]. At the beginning of the development process, the focus was on both analysis of the learning objectives of the existing tutorials and the search for a suitable exchange platform through which learning content could be conveyed and its correct implementation ensured.

Subsequently, four topics were selected and developed as digital tutorials: “Placement of a bladder catheter”, “Placement of an intravenous peripheral cannula”, “Evaluation of an electrocardiogram”, and “Practice of surgical suturing and knotting techniques”. In terms of teaching methodology, the flipped classroom approach [9] formed a common basis for all digital tutorials in terms of the targeted linking of asynchronous and synchronous content. In addition, the tutorials were designed according to Peyton's 4-step method [10]: first, the correct application was demonstrated in videos, followed by deconstruction (and reconstruction) of an improvised model. Finally, once the PTs had confirmed their understanding, the procedure was carried out. The factual knowledge was imparted to the PTs in advance via partly self-produced content (e.g. video tutorials, literature, quizzes) on the Moodle learning management platform. Afterwards, the PTs met with the TUTs for a virtual meeting in order to clarify open questions and to practice under supervision. The successful small-group approach, with up to eight PTs [11] from the in person tutorials, was retained. Within the digital format, the PTs created a model surrogate at home, on which they practiced with centrally distributed materials. It was not possible to issue training models due to their insufficient number and for reasons of hygiene.

## Concept demonstration – example tutorial: “insertion of a bladder catheter”

**Figure 1:**
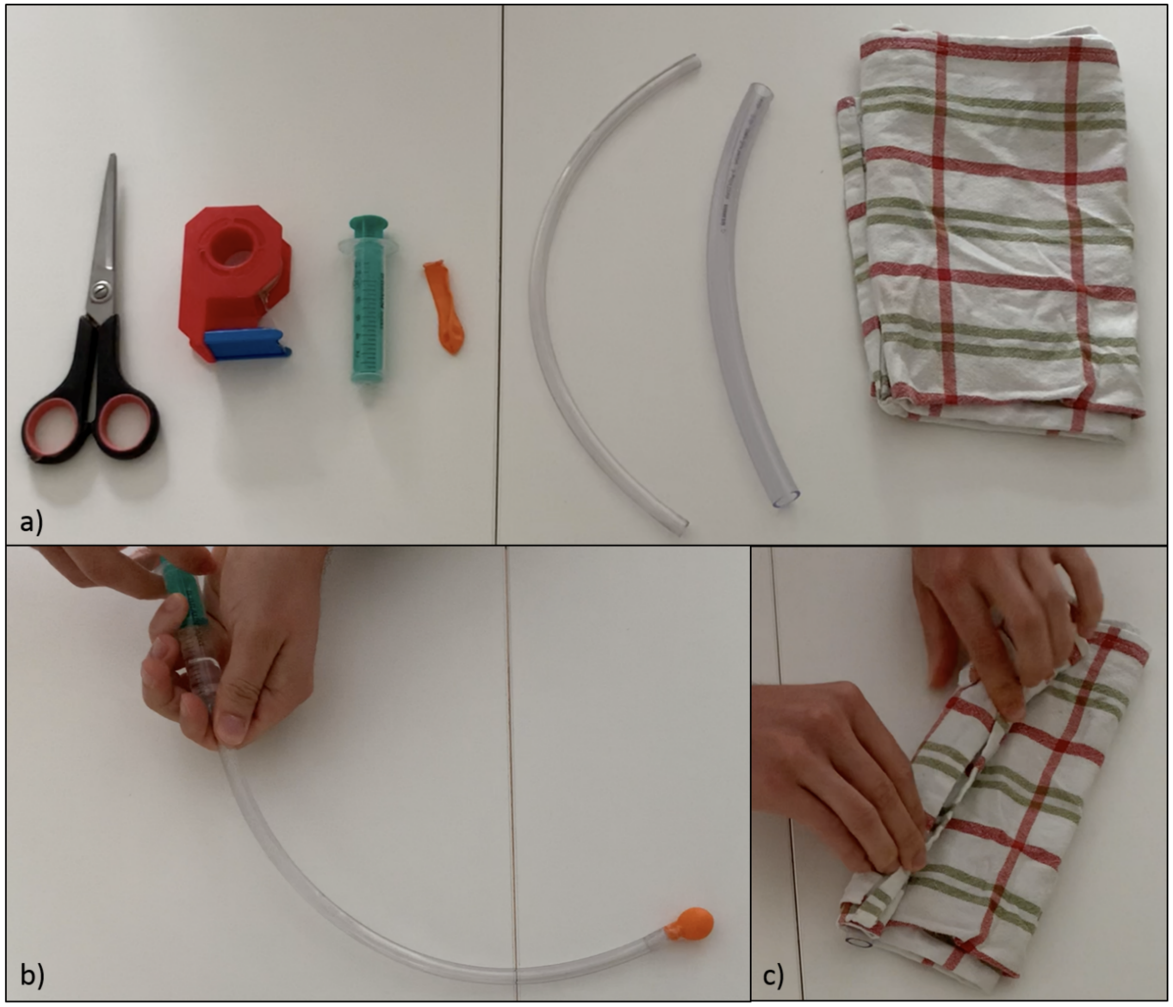
Instructions for assembling catheter and urethra a) shows the materials needed to assemble the catheter and penile shaft at home. The tubes have two different diameters. b) The catheter can be assembled at home with a tube, a balloon and adhesive tape. To test if the catheter is leak-proof, it is blocked with a disposable syringe filled with water. c) For the simulated penis shaft, the tube (larger diameter than catheter) is wrapped with a tea towel and this is fixed with adhesive tape.

**Figure 2:**
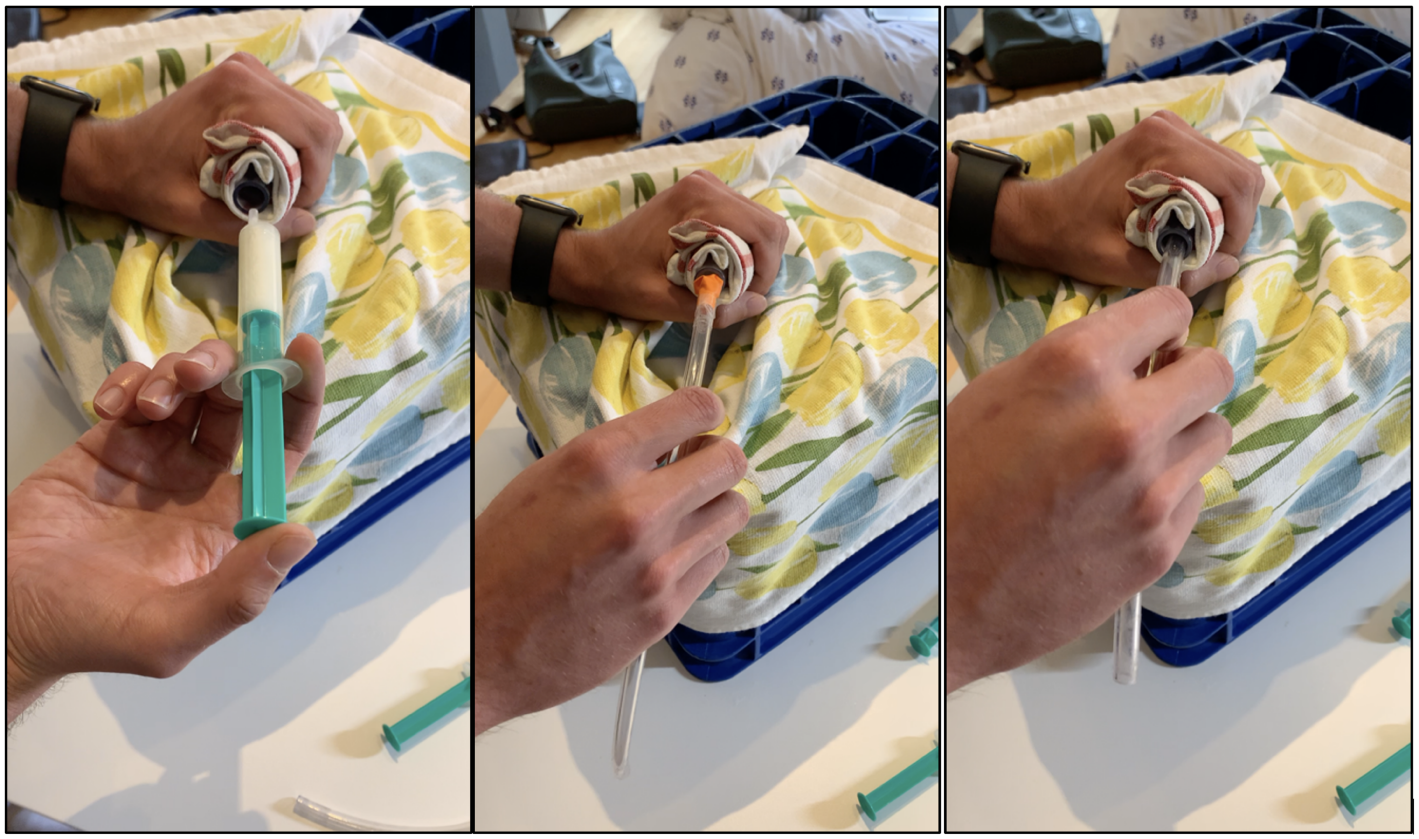
Catheterization of the improvised bladder catheter model by a PT.

In the in-person tutorial, the “insertion of a bladder catheter” was practiced on a model. The focus of the digital tutorial was initially on the TUTs' innovative, supervised demonstration of the urethra and bladder using everyday items to simulate their complex anatomy (see figure 1 (Fig. 1)). The simulation was supported by different training videos [12], specially designed by the TUTs and made available via Moodle. Following this, the individual steps in the sterile “insertion of a bladder catheter” were practiced in a virtual meeting under supervision and observation by the TUT (see figure 2 (Fig. 2)). The PTs thus received direct feedback and were thus armed with the skills to avoid mistakes identified by the TUT. 

## Implementation

Following the announcement of the teaching offer via the faculty-internal platform mediTUM, further communication took place directly between PTs and TUTs: the PTs established personal contact via e-mail. They were then registered on the Moodle course and, after arranging an appointment for a virtual meeting, were able to pick up the exercise materials at a central location.

So far, digital tutorials have been conducted on 23 dates since July 2020, which were attended by 64 PTs (M=2.8, min 1, max 7). In particular, the tutorials with only 1 PT presented the TUT with the challenge of having to assume two roles at the same time, i.e., both to exchange information about their own experiences and teach competencies.

## Acceptance

All tutorials conducted were reviewed and optimized for quality with the help of an online questionnaire-based evaluation (see table 1 (Tab. 1)). The 24 completed questionnaires reflected an overall positive response: on a scale of 1 (“do not agree at all”) to 6 (“agree completely”), the average rating was 5.2. The PTs rated both training delivery and their own progress as “good” to “very good”; it was only in the area of independent performance of the respective procedure on patients that the PTs felt only moderately well-prepared. The optional free text responses revealed a clear appreciation of the instruction offer as well as the implementation by the TUTs.

## Further development & conclusion

Starting from the winter semester 2020/21, in-person tutorials can again take place in compliance with the hygiene regulations of the Faculty of Medicine at the Klinikum rechts der Isar, see [https://www.lehren.tum.de/downloads/]. The long-term plan, even once the pandemic has passed, is to combine them with the digital tutorials. For example, the Moodle course could be serve as the flipped classroom by providing information materials and forums as theoretical preparation for practical application during in-person tutorials [13]. In addition, the improvised models can be used during in-person tutorials to map more parallel practical practice. From the free text responses in the evaluation of the digital format, it is clear that the PTs would like to have the opportunity to work on the models at the simulation center. The fact that there were 278 registrations within a few hours for the 72 slots available for the very limited 14 in-person appointments reflects the high level of student need for physical presence on the courses. The offer of digital appointments is well-accepted and appreciated by students, but not nearly to the same extent.

The hybrid form of the course now envisaged should be assessed in terms of effectiveness and sustainability as part of accompanying research with the initial presence-only event. It is already clear that the still fledgling student tutorial program “How to... train your skills!” has undergone significant and promising further development. This is the result not only of the combination of digital and in-person tutorials, but also of the situational change of perspective and being constrained to apply practical skills at a distance and with the simplest improvised means.

## Competing interests

The authors declare that they have no competing interests. 

## Figures and Tables

**Table 1:**
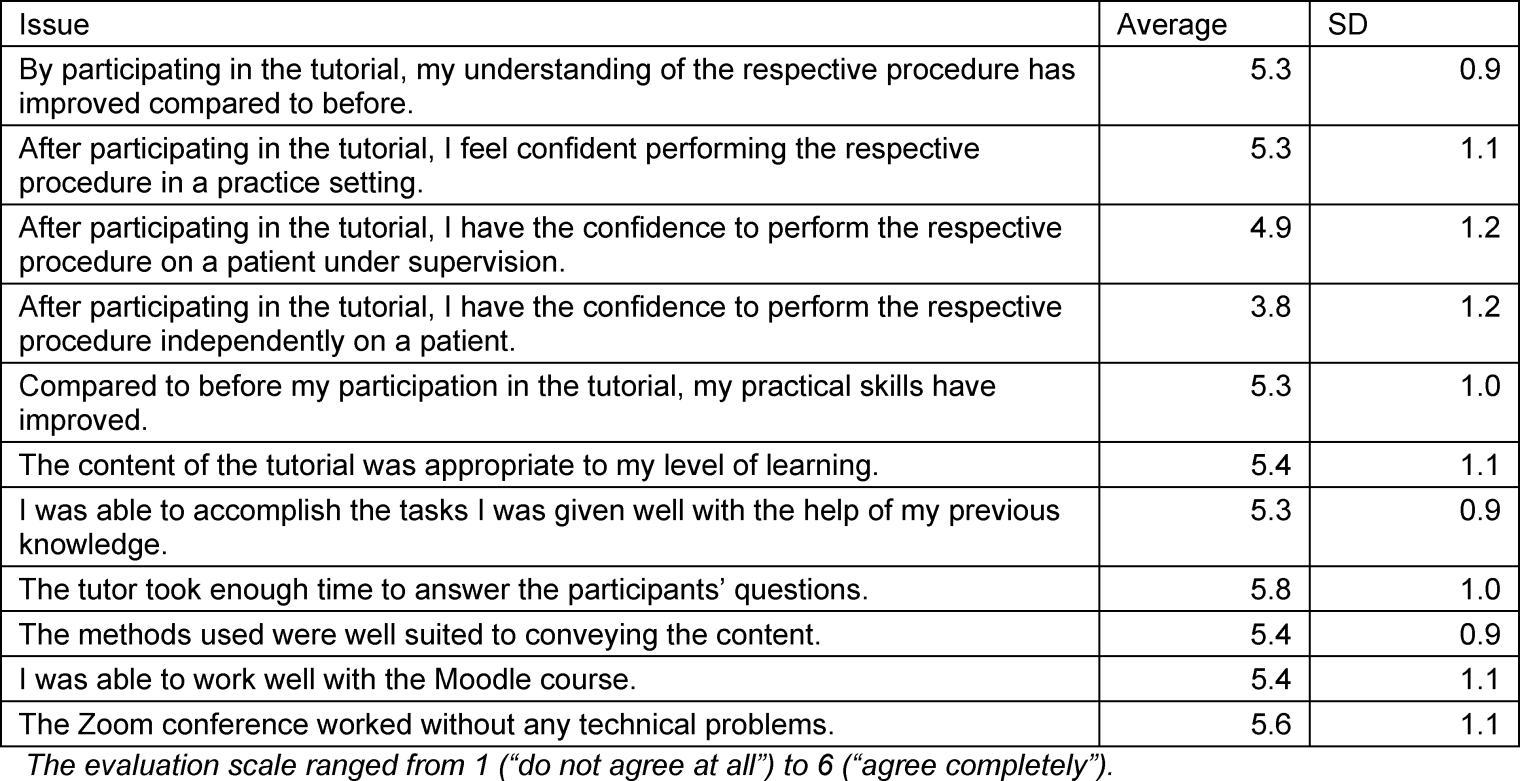
Evaluation of the digital tutorials conducted to date (n=24)
